# Detection of hotspots of school dropouts in India: A spatial clustering approach

**DOI:** 10.1371/journal.pone.0280034

**Published:** 2023-01-17

**Authors:** Raghul Gandhi Venkatesan, Bagavandas Mappillairaju

**Affiliations:** 1 Department of Mathematics, SRM Institute of Science and Technology, Chengalpattu, Tamil Nadu, India; 2 Centre for Statistics, SRM Institute of Science and Technology, Chengalpattu, Tamil Nadu, India; Zoological Survey of India, INDIA

## Abstract

School dropout is a significant concern universally. This paper investigates the incorporation of spatial dependency in estimating the topographical effect of school dropout rates in India. This study utilizes the secondary data on primary, upper primary, and secondary school dropout rates of the different districts of India available at the Unified District Information System for Education plus (UDISE+) for the year 2020 to contemplate the impact of these dropouts from one region to different regions in molding with promotion rate and repetition rate. The Global Moran’s I, Univariate and Bivariate Local Indicators of Spatial Association, and spatial models are utilized to investigate the geographical variability and to find the possible relationship between dropout rates and the school-level factors at the district level. The outcomes provide clear spatial clustering and precisely highlight the hot zone dropout regions with high repetition and low promotion rates. Based on this study’s results, educational administrators can make evidence-based decisions to reduce dropout rates in hot zones of various regions of India. Furthermore, futuristic studies focusing on linking spatial hot zones with causal factors will add consistent data in assisting policymakers in taking necessary measures to develop a sound education management system.

## Introduction

Education is a foundation for human progress toward creating a healthy society. Its effects are significant in the development of individuals and the whole country. India’s school education vision 2030 intends to qualitatively improve the nation’s current educational system and provide high-quality education to all children of the school-attending age group, whose numbers are estimated to climb from 25 crores in 2010 to 30 crores in 2030. Though the school enrolment rates have increased in the past few years, the percentage of students who drops out of school has either remained the same or increased. As per UDISE+ 2020 report, the dropout rate in the secondary level (17%) is still high compared to the primary (1.8%) and upper primary level (1.5%). This percentage of school dropouts adds a quantitative inclusion burden to India’s vision of establishing education goals [1].

The Dissimilarity in the primary education curriculum and school infrastructure has been noted to play a key role in primary education outcomes. As a result, the government executed a new policy named "universalization of elementary education" (UEE) in 2001. UEE focuses on three major elements: universalization, which guarantees that all students between the ages of 6 and 14 have direct exposure to a school; enrolment universalization, which guarantees that all students in the aforesaid age are enrolled in school; and retention universalization, which guarantees that students who started primary school progress till they complete the upper primary level [2, 3].

In the continuum of the retention universalization goal, research on factors influencing school dropout generally concentrates on child, family, school and community-related factors [4–8]. Only a few studies incorporated the regional variation in the proportion of dropout rate [9]. The use of geographic information systems in educational research, planning, and policy strategizing is a relatively recent development. However, it is becoming more common as researchers realise the benefits of providing a visual depiction of statistical data [10]. A recent study from India used spatial techniques to identify district-level variation between education programs and literacy rates based on the first major element of UEE. School dropouts remarkably increase quantitative content to the third major element of UEE—retention universalization [11]. Universally, Jose Eos Trinidad (2022) used spatial tools to analyze the determinants of high school dropout with race and poverty in New York City [12]. Mark. J. Schafer (2006) performed school-level spatial analysis to determine the relationship between school-level factors and high school dropout in Louisiana [13]. In India, Our study is the first to examine the regional variability and school-level risk factors of primary, upper primary and secondary dropouts across all the districts of India. District-level promotion rate and repetition rate of boys and girls are the School level risk factors examined in this study for possible impact on dropout. Each district is chosen as the analytical unit and used as a spatial region to highlight the regional variations in dropout and school-level factors in contemplation to carefully explore the relationships adjusting the potential covariates resulting from geographical influences.

To improve the efficiency of the school management framework, this approach incorporating geospatial technology will assist policy-makers and researchers in better understanding the existing status. Additionally, this would support the Sarva Shiksha Abhiyan and National Education Policy in providing direction for formulating preventive measures to decrease dropouts. The spatial representations of education policies across districts can help to guarantee that every student in India completes their schooling at any cost to enhance their quality of life. Policy-makers and government representatives can also utilize findings from this study to improve the current policies, fostering India’s school education and assisting in developing, testing, and implementing cost-effective strategies in hotspot regions to reduce dropouts in India.

## Material and methods

### Data

The UDISE+ of India recently released data for the year 2020, accessible on the UDISE+ website https://dashboard.udiseplus.gov.in. The reports have been published under the Department of School Education and Literacy (DoSEL), Ministry of Education, Government of India. This report is based on data that schools with active UDISE+ codes in a reference year voluntarily uploaded using a data collection format (DCF) specifically created for this report. The State/UT government of the school’s location assigns the UDISE+ code for institutions. The District Education Officer (DEO) at the district level ensures that the information entered into the DCFs is accurate. This report offers essential information on several factors, such as the number of schools, teachers, and students who were enrolled, promoted, and dropped out, in terms of counts and percentages. This data source is the input for the analysis [1].

### Response and predictors

Following the conceptual frameworks of earlier studies [12–14], the district-level overall dropout rates for primary, upper primary, and secondary levels were the outcome variables considered in this study. Utilizing the aggregate district-level data, this study included six independent variables: promotion rate, repetition rate, and a dropout rate of boys and girls.

### India digital map

The primary researcher downloaded India’s district-level base map from kaggle at https://www.kaggle.com/datasets/raghulgandhi/indian-district-map-726. Initially, the number of districts reported in UDISE+ was 733. However, a few districts in Arunachal Pradesh, Delhi, Karnataka, Manipur, Tamil Nadu, and West Bengal were merged for analysis purposes using their boundary, and the number of districts considered for the analysis was 726 given in S1 Dataset.

### Analysis

To examine the geographic distribution of dropouts in primary, upper primary, and secondary levels in India, several quartile maps at the district-level were created. To investigate the geographical correlation and grouping of districts, Moran’s I, LISA cluster map, and significance maps were created. To calculate the distance in space between every potential pair of observable units in the dataset. The Queens’ contiguity approach has been used to produce the spatial weight matrix of order one [15]. Since the regional coordinates and attribute data on school dropouts are discontinuous, no clear spatial relationship exists. According to Queens’ technique, neighbors are geographical districts with a non-zero length shared border. The Moran’s I statistic reveals how identical or distinct observed values are to their geographical neighbors [16]. Therefore, values for the univariate and bivariate LISA were generated to examine the spatial correlation of the dropout rates among districts. The Moran’s I statistic can be calculated using the following formula [17]:

$$\text{Univariate}\;\text{Moran}’\text{s}\;\text{I}=\frac{n}{S_{0}}*\frac{\sum_{m}\sum_{n}W_{mn}\left(y_{m}-\bar{Y}\right)\left(y_{n}-\bar{Y}\right)}{\sum_{m}{\left(y_{m}-\bar{Y}\right)}^{2}}$$ (1)

Where y denotes the dropout and $\bar{y}$ denotes the average of y; n denotes the number of districts; *W*_*mn*_ denotes the standardised weight matrix connecting observation m and n; and *S*_0_ denotes the total of all geographical weights.

$$\text{Bivariate}\;\text{Moran}’\text{s}\;\text{I}=\frac{n}{S_{0}}*\frac{\sum_{m}\sum_{n}W_{mn}\left(y_{m}-\bar{Y}\right)\left(z_{n}-\bar{Z}\right)}{\sum_{m}{\left(z_{m}-\bar{Z}\right)}^{2}}$$ (2)

Where y and z represent the dropout and predictors; $\bar{Y}$ represents the average of y; $\bar{Z}$ represents the average of predictors; n denotes the number of districts; *W*_*mn*_ denotes the standardised weight matrix connecting observation m and n; and *S*_0_ denotes the sum of all geographical weights.

A positive spatial autocorrelation suggests that spots with identical data points are strongly connected in the area, while a negative spatial autocorrelation shows that strongly connected spots are more distinct. The values of Moran’s I typically range from (-1, 1), with positive measures indicating the geographical grouping of comparable measures and negative measures indicating the spatial grouping of different measures. In the absence of any spatial autocorrelation, a measure of 0 indicates a random geographical distribution. The association of nearby values around a particular geographic region is measured by univariate LISA [17]. It establishes the degree of spatial grouping and unpredictability that the data exhibit [18, 19].

The following formula provides the measure *I*_*i*_:

$$I_{i}=\frac{n.\left(y_{m}-\bar{Y}\right)}{\sum_{m}{\left(y_{m}-\bar{Y}\right)}^{2}}*\sum_{n}w_{mn}\left(y_{n}-\bar{Y}\right)$$ (3)

Four different types of autocorrelations were identified based on the Moran’s scatter plots and are referred to as:

- Districts with high measures and identical neighbouring districts are known as “hot spots” (High-High).
- Districts with low measures and identical neighbouring districts are known as “cold spots” (Low-Low).
- Districts that are high in measures but have low-measure neighbouring districts (High-Low) and districts that are low in measures but have high-measure neighbouring districts (Low-High) are known as “Spatial Outliers”.

In the same way, bivariate LISA were also calculated to examine the relationship between the repetition rate and promotion rate of both boys and girls of regions with different dropout rates.


$$I_{i}=\frac{n.\left(y_{m}-\bar{Y}\right)}{\sum_{m}{\left(z_{m}-\bar{Z}\right)}^{2}}*\sum_{n}w_{mn}\left(y_{n}-\bar{Y}\right)$$ (4)

LISA cluster and significance map were produced in the Geo-Da by utilizing the LISA tools. The map shows the districts with significant Moran’s I value categorized in terms of spatial autocorrelation, where hotspots are defined by red, coldspots by deep blue, and spatial outliers by light blue and light red. To investigate the possible relationships between the dropouts and predictors, we carried out statistical regressions. We first used the ordinary least square (OLS) model, then we calculated the spatial autocorrelation in the OLS regression residuals to check the spatial heterogeneity caused by spatial dependency. As soon as we determined that the Moran’s I statistic for each of the outcomes was statistically significant, we calculated the spatial lag model (SLM) and spatial error model (SEM) to obtain unbiased measures of the correlations between the predictors and dropout while addressing the geographical heterogeneity that existed in the data. A standard SLM assumes that the data points are spatially dependent and lag to one another in the nearby regions, In contrast, the SEM assumes that the disturbance terms are correlated with nearby geographical units. The best model was then determined by comparing the Akaike Information Criterion (AIC) and Schwartz Criterion (BIC) values, and we observed that SEM provided the better fit for this particular study.

The fundamental formula for OLS is as follows:

Y=α+βX+ε (5)

Where Y is the response, x denotes the predictors’ vector, α is the model’s intercept and β is the associated coefficient vector. The assumption is that the error component (ε) is identically and independently distributed (i.i.d). If it is found that there is a significant spatial dependency, simultaneity bias in the OLS likely result in erroneous and inaccurate estimations of the model indicators. However, SLM and SEM models are fitted as follows to limit the topographical effects.

SLM, if the response variable (Y), is correlated to the weighted mean of the observations in its surrounding regions, where ρ is the auto-regressive parameter, then

$$Y=\rho w_{y}+\beta x+\varepsilon$$ (6)

SEM, if residuals reveal spatial dependency, the subsequent model effectively manages the spatial effect.


$$Y=\beta x+\varepsilon ,\;\text{where}\;\varepsilon =\lambda w_{\varepsilon }+\zeta$$ (7)

Here, λ denotes the auto-regressive parameter; *ζ* is the i.i.d. disturbance term. By increasing the relevant likelihood functions, both SEM and SLM are estimated [13]. QGIS desktop 3.26.2 and GeoDa 1.20.0.8 software were used for the statistical analysis.

## Results

Table 1 gives the nation’s overall dropout rate, promotion rate, and repetition rate. Results show that the secondary dropout rate is the biggest concern than the upper primary and primary dropout rates in India. The summary of the predictors shows that 99 percent of students at the primary level, 97 percent of students at the upper primary level and 84 percent of students at the secondary level were promoted. In the case of the repetition rate, 0.51 percent of students at the primary level, 0.56 percent of students at the upper primary level and 1.85 percent of students at the secondary level repeated their grades in India.

**Table 1:** Rates of promotion rate, repetition rate, and dropout rate in India-2019-2020.

Dependent Variables	Percentage
Primary Dropout Rate-Overall	0.76
Upper Primary Dropout Rate-Overall	2.27
Secondary Dropout Rate-Overall	14.04
**Independent Variables (Predictors)**	
Primary Promotion Rate-Boys	98.66
Primary Promotion Rate-Girls	98.8
Primary Repetition Rate-Boys	0.51
Primary Repetition Rate-Girls	0.51
Primary Dropout Rate-Boys	0.83
Primary Dropout Rate-Girls	0.69
Upper Primary Promotion Rate-Boys	97.5
Upper Primary Promotion Rate-Girls	96.82
Upper Primary Repetition Rate-Boys	0.55
Upper Primary Repetition Rate-Girls	0.57
Upper Primary Dropout Rate-Boys	1.95
Upper Primary Dropout Rate-Girls	2.61
Secondary Promotion Rate-Boys	83.77
Secondary Promotion Rate-Girls	84.48
Secondary Repetition Rate-Boys	1.89
Secondary Repetition Rate-Girls	1.81
Secondary Dropout Rate-Boys	14.34
Secondary Dropout Rate-Girls	13.71

### Spatial pattern and clustering of school dropouts across India

**Fig 1:**
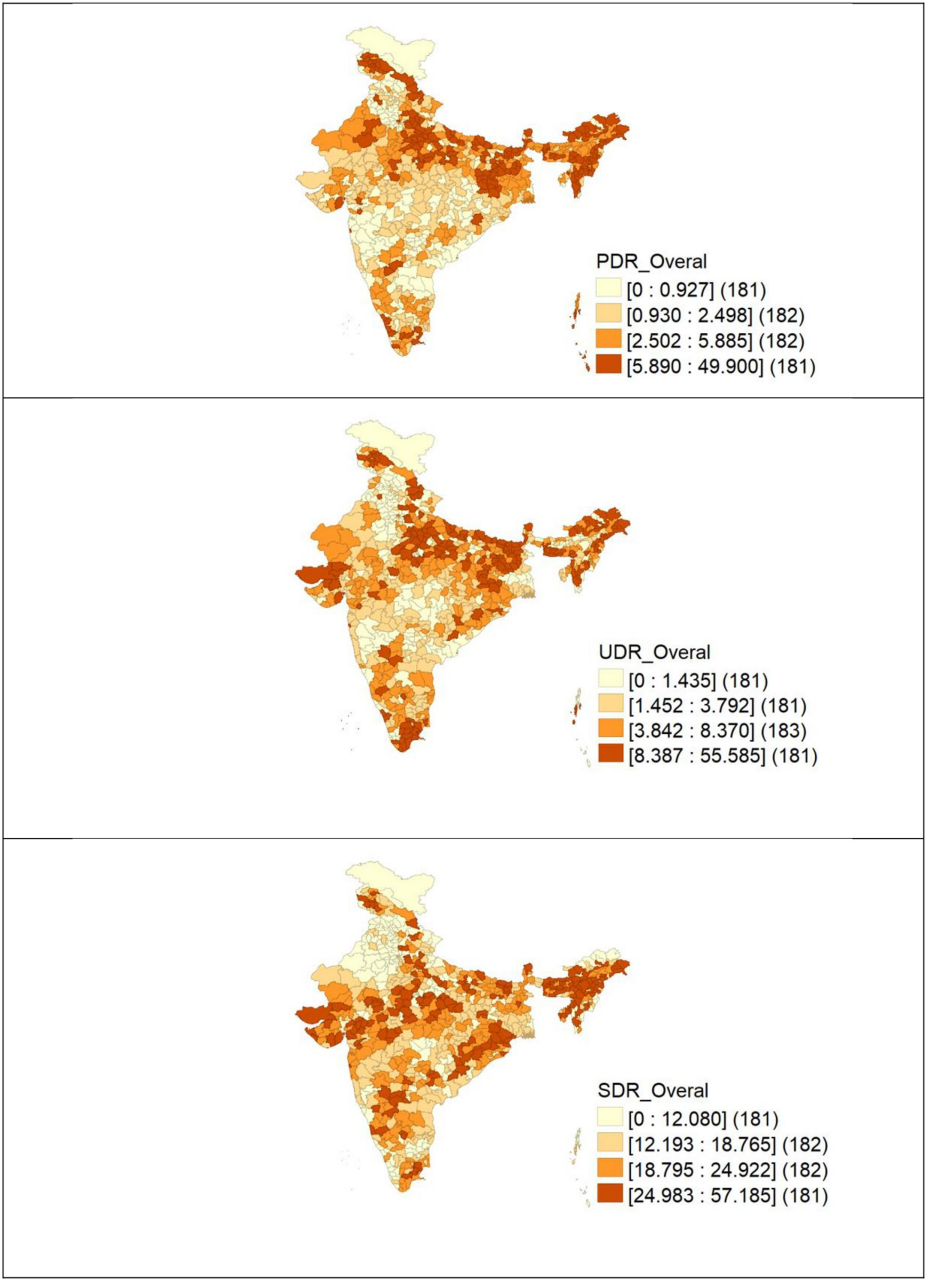
Rates of dropout across Indian districts. (A) Primary dropout. (B) Upper primary dropout. (C) Secondary.

The spatial distribution of districts’ primary, upper primary and secondary dropout rates is depicted in Fig 1A–1C. The color highlights the geographical variations in dropout rates. The lighter color represents a lower dropout rate, whereas the darker color represents a greater dropout rate in certain districts. From the spatial maps, it is obvious that dropout rates vary geographically throughout the districts.

**Fig 2:**
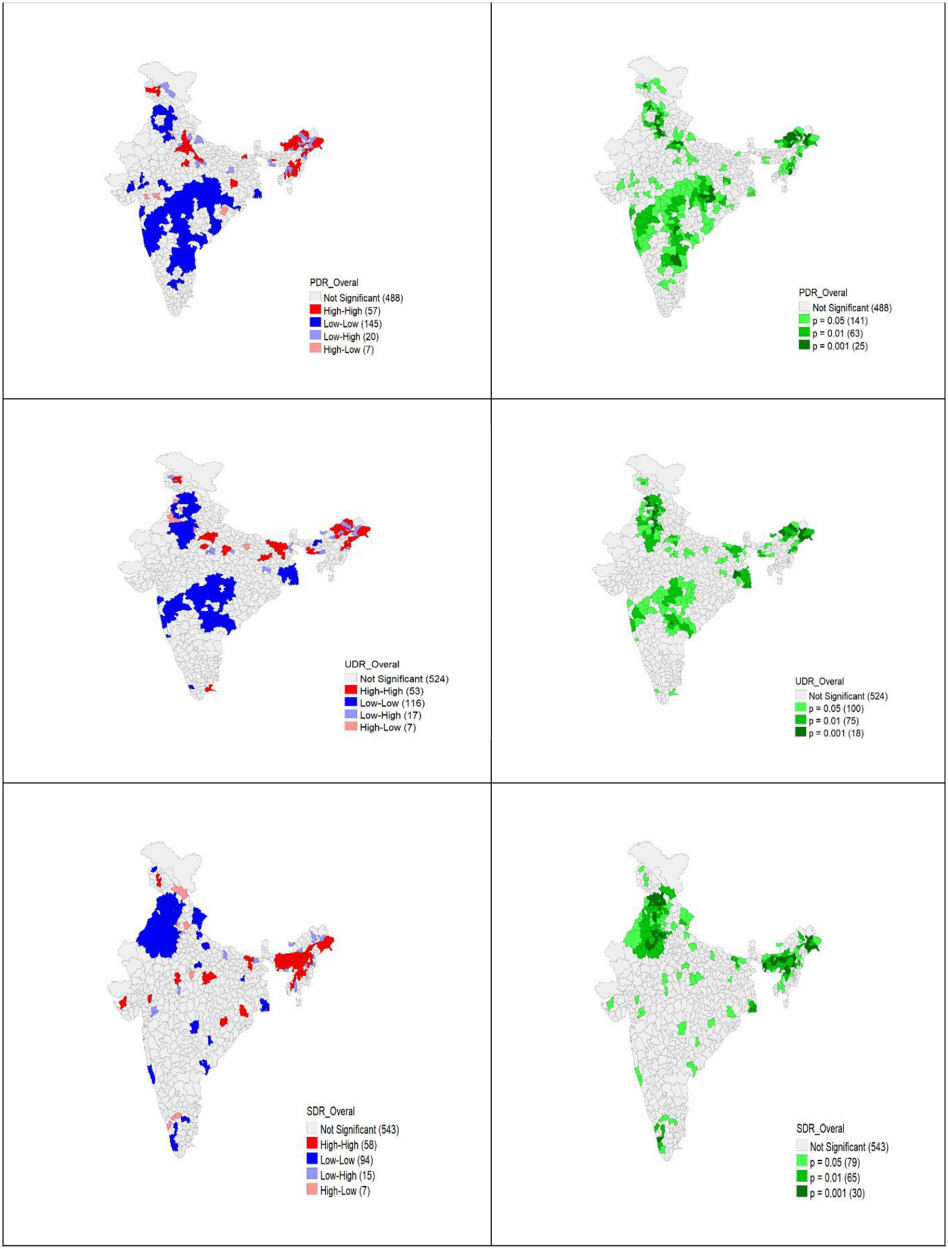
LISA cluster and significance maps of dropout rates across Indian districts. (A) LISA map of primary dropout rate (I = 0.349, <0.05). (B) Significance map of primary dropout. (C) LISA map of upper primary dropout rate (I = 0.367, <0.05). (D) Significance map of upper primary dropout. (E) LISA map of secondary dropout rate (I = 0.316, <0.05). (F) Significance map of secondary dropout.

The estimated values of the Moran’s I statistic shows primary, upper primary and secondary dropout rates across the districts have spatial autocorrelation. The LISA cluster map in Fig 2A and the significance map in Fig 2B show 57 hotspots in primary dropout across the Indian districts. Similarly, Fig 2C shows 53 hotspots in upper primary dropout in Indian districts. In secondary dropout, 58 hotspots were identified and are shown in Fig 2E. The number of districts in each state which are hotspots is given in Table 2 and the S1 Appendix gives Moran’s, I statistic, cluster number and p-value for all 726 districts.

**Table 2:** District-level hotspots of school dropouts.

State	Primary Dropout Hotspot	Upper Primary Dropout Hotspot	Secondary Dropout Hotspot
**Arunachal Pradesh**	14	13	4
**Assam**	4	2	28
**Bihar**	1	13	4
**Gujarat**	0	0	2
**Jammu & Kashmir**	7	5	3
**Jharkhand**	2	1	0
**Madhya Pradesh**	0	1	3
**Manipur**	8	0	1
**Meghalaya**	1	4	6
**Mizoram**	1	0	0
**Nagaland**	4	5	3
**Odisha**	0	0	2
**Tamil Nadu**	0	1	0
**Tripura**	1	0	2
**Uttar Pradesh**	14	8	0

The estimated values of bivariate LISA shown in Table 3 indicate the results of the spatial relationship between the primary, upper primary and secondary dropout rate with the other predictors taken in this study. Among the various predictors, the promotion rate of boys and girls consistently showed a negative, dropout rate of boys and girls showed a positive spatial autocorrelation at the 5% level of significance with primary, upper primary and secondary dropout across the districts. In the case of Upper primary dropout, the repetition rate of boys and girls showed positive spatial autocorrelation at the 5% level of significance. In the case of secondary dropout, boys’ repetition rate showed a negative spatial autocorrelation at significance level (<0.05) and girls’ repetition rate showed very low (insignificant) spatial autocorrelation.

**Table 3:** Bivariate spatial association between dropouts and their predictors.

Predictors	Primary Dropout	Upper Primary Dropout	Secondary Dropout
**Boys Promotion Rate**	-0.272(0.001)	-0.297(0.001)	-0.200(0.001)
**Girls Promotion Rate**	-0.267(0.001)	-0.309(0.001)	-0.219(0.001)
**Boys Repetition Rate**	0.106(0.001)	0.147(0.001)	-0.037(0.001)
**Girls Repetition Rate**	0.089(0.001)	0.157(0.001)	0.009(0.331)
**Boys Dropout Rate**	0.31(0.001)	0.32(0.001)	0.302(0.001)
**Girls Dropout Rate**	0.289(0.001)	0.327(0.001)	0.302(0.001)

Each cell shows the corresponding value of bivariate Local Moran’s I statistic and the p-value

### Estimated outcomes from the OLS, SLM and SEM models

Table 4 presents findings from OLS, SLM, and SEM models that describe how factors affect different dropouts after controlling for topographical effects. Based on the model selection criteria, SEM was observed to be the better-fitted model for all primary, upper primary, and secondary dropouts.

**Table 4:** OLS, SLM and SEM estimation of school dropout, India.

Predictors	Primary dropout			Upper Primary dropout			Secondary dropout		
	OLS	SLM	SEM	OLS	SLM	SEM	OLS	SLM	SEM
Boys Promotion Rate	0.103 (0.001)	0.103 (0.001)	0.103 (0.0001)	-0.255 (0.001)	-0.255 (0.001)	-0.255 (0.001)	0.059 (0.001)	0.059 (0.001)	0.059 (0.001)
Girls Promotion Rate	-0.113 (0.001)	-0.113 (0.001)	-0.110 (0.001)	0.245 (0.001)	0.245 (0.001)	0.245 (0.001)	-0.075 (0.001)	-0.076 (0.001)	-0.075 (0.001)
Boys Repetition Rate	0.024 (0.83)	0.024 (0.84)	0.071 (0.53)	-0.354 (0.001)	-0.356 (0.001)	-0.364 (0.001)	-0.101 (0.01)	-0.103 (0.01)	-0.102 (0.01)
Girls Repetition Rate	-0.074 (0.57)	-0.074 (0.56)	-0.089 (0.48)	0.327 (0.001)	0.332 (0.001)	0.336 (0.001)	0.099 (0.03)	0.100 (0.03)	0.101 (0.02)
Boys Dropout Rate	0.604 (0.001)	0.603 (0.001)	0.602 (0.001)	0.229 (0.001)	0.230 (0.001)	0.229 (0.001)	0.624 (0.001)	0.625 (0.001	0.623 (0.001)
Girls Dropout Rate	0.418 (0.001)	0.418 (0.001)	0.422 (0.001)	0.785 (0.001)	0.785 (0.001)	0.785 (0.001)	0.377 (0.001)	0.377 (0.001)	0.378 (0.001)
Number of observation	726	726	726	726	726	726	726	726	726
R square	0.9673	0.9673	0.9682	0.9723	0.9723	0.9723	0.9745	0.9745	0.9745
AIC	2428.53	2430.42	2415.43	2405.81	2407.64	2405.63	2723.74	2725.64	2722.45
BIC	2460.65	2467.12	2447.55	2437.92	2444.34	2437.74	2755.85	2762.34	2754.56

OLS-Ordinary Least Square Model, SLM-Spatial Lag Model, SEM-Spatial Error Model

Each cell gives the estimated coefficients from the regression models and the p-value.

#### Primary dropout

The estimated coefficients of OLS regression were 0.103(<0.05) for boys’ promotion rate, -0.113(<0.05) for girls’ promotion rate, 0.604(<0.05) for boys’ dropout rate, 0.418(<0.05) for girls dropout rate were statistically significant to primary dropout. After making spatial modifications using the spatial model, it was observed that the pattern of the relationship between predictors and primary dropouts remained the same. We found SEM (2415.43, 2447.55) as the best fit since it has the lowest AIC and BIC values when comparing SLM (2430.42, 2467.12) and OLS (2428.53, 2460.65).

#### Upper primary dropout

The estimated coefficients of OLS regression were -0.255(0.001) for boys’ promotion rate, 0.245(0.001) for girls’ promotion rate, -0.345(0.001) for boys’ repetition rate, 0.327(0.001) for girls’ repetition rate, 0.229(0.001) for boys dropout rate, 0.785(0.001) for girls dropout rate were statistically significant to upper primary dropout. After making spatial modifications using the spatial model, it was observed that the pattern of the relationship between predictors and upper primary dropouts remained the same. The corresponding AIC and BIC values of OLS (2405.81, 2437.92), SLM (2407.64, 2444.34) and SEM (2405.63, 2437.74) are obtained and SEM is considered to be the best fit.

#### Secondary dropout

The estimated coefficients of OLS regression were 0.059(0.001) for boys’ promotion rate, -0.075(0.001) for girls’ promotion rate, -0.101(0.001) for boys’ repetition rate, 0.099(0.001) for girls repetition rate, 0.624(0.001) for boys dropout rate, 0.377(0.001) for girls dropout rate were highly significant to secondary dropout. After making spatial modifications using the spatial model, it was observed that the pattern of the relationship between predictors and secondary dropouts remained the same. We found SEM (2722.45, 2754.56) as the best fit since it has the lowest AIC and BIC values when comparing OLS (2723.74, 2755.85) and SLM (2725.64, 2762.34).

## Discussion

School graduation denotes promotion from primary to secondary. Hence high promotion rate yields a collective benefit in aligning towards the retention universalization policy of UEE, propelling the whole education system processing towards India’s vision for 2030. Our study, based on geospatial techniques, reveals that School dropouts are one of the essential criteria for increasing a country’s overall school graduation percentage. Hence, dropping out is an issue that has to be solved [20]. Researchers have examined potential influences and identified significant effects of child, family, school, and community factors [21–25]. In addition to these specific concerns, the impact is even worse when child and family factors such as sex, caste, and religion combine with school-level factors such as attendance, pupil-teacher ratio, and school infrastructure [9, 26, 27]. An additional list of objects that are rarely researched is spatial factors.

The current study intends to apply the existing method for assessing the geographical neighborhood factors that influence dropout incidence and to evaluate significant findings on these factors in India. We emphasize that spatial statistics can give valuable information for analyzing dropout distribution and point out its importance.

With the use of the district-level information from the UDISE+ India of 2020, a LISA cluster map is generated. Based on the thematic maps, we observed that district dropout rates were not simply random. In particular, several districts in Arunachal Pradesh, Assam, Bihar, Gujarat, Jammu & Kashmir, Jharkhand, Madhya Pradesh, Manipur, Meghalaya, Mizoram, Nagaland, Odisha, Tamil Nadu, Tripura, and Uttar Pradesh are considered to be hotspots for dropouts in the primary, upper primary and secondary levels because these clusters of neighborhoods have a low rate of promotion and a high rate of repetition.

Further investigation on hotspots reveals a correlation between the dropouts and the promotion rate and repetition rate of boys and girls. Although previous studies have linked child and family factors to a higher risk of dropout [28], the current study emphasises that districts with high repetition rates and low promotion rates may also impact dropout rates. Districts in Arunachal Pradesh, Jammu & Kashmir, Nagaland, Assam, and Bihar have stood out, particularly in these hotspots. Even though dropout rates are greater in low promotion and high repetition rate districts, this does not imply that dropout rates are also higher in these regional clusters. There are several districts where the dropout and promotion rates are inversely proportional. In addition to the few co-locational clusters, low promotion rates, and high repetition rate districts also have outliers in terms of dropout rate. So, Researchers must be cautious when making conclusions. They must also qualitatively explore why certain nearby districts have noticeably different dropout rates despite having a nearly identical promotion and repetition rates. It is feasible that the dropout rate might be less due to the standard initiatives implemented between blocks at the district level [29].

Despite being focused on Indian districts, this study’s methodology may also be employed at the block level. Understanding the spatial features and clusters of districts with greater block-level dropout rates is more required than merely identifying the districts with the highest dropout rates. In this regard, geospatial studies can assist in identifying block-level dropout hotspots to help make decisions that possess the capacity in changing district-level dropout trends.

In this study, we emphasized the importance of spatial analysis of district-level dropout rates using quartile, univariate-bivariate LISA, and spatial autoregressive models. It is possible to find the district-level variations of dropout rates using quartile maps. Clustered LISA maps displayed districts with comparable high or low dropout rates close to one another. This identification of dropout hotspots or coldspots can increase the effectiveness of contextualized interventions [30]. LISA maps highlight outliers, and finding outliers might encourage the qualitative researcher to investigate what would make a specific district differ from its neighboring districts. Additionally, spatial regressions were employed in this study to investigate the possible correlation between the dropout and promotion-repetition rates of boys and girls.

Although this study contributes some conceptual and methodological insights, it has certain limitations. Firstly, GIS-based research will always have difficulty with data availability, and this study also faced the same. Second, this study analyzed the pattern of district-level dropout rates and their influencing school-level characteristics across districts within a district-level framework. This analysis might be taken further and applied to block levels to determine the intra-district variation in student dropout rates, which could help to determine school-level factors influencing the dropout variation within districts. Third, though this study examined the regions in India with the highest dropout rates, our study only focused on spatial factors and not the causative framework. Fourth, we exclusively considered the characteristics at the school level specific to the promotion-repetition rates and excluded other factors related to child, family and community. This study is the first attempt to use spatial analysis for dropout data at the district level in India, therefore this limitation offers an opportunity for future work to expand the knowledge about how certain factors might be geographically clustered and examine regional patterns.

Despite its limitations, this study highlights the conceptual findings to advance our understanding of how dropout rates are influenced by geographical locations and methodological strategies for using GIS tools to investigate educational sector challenges. The inference that dropout rates in India are geographically grouped in specific districts in the north-eastern and central states is thematically supported by the evidence. These factors are not deterministic, although high dropout rates are correlated with low promotion rates and high repetition rates.

This study recommends and supports the use of GIS approaches that are suitable for concerns and challenges in education. Such techniques can enhance conceptual knowledge and practical implications by identifying spatially effective remedies. These conceptual and methodological contributions may motivate the researchers to explore the methodology for real-world problems including strategic planning, policy-making, and decision-making in the education sector.

## Conclusion

Although school enrolment in India has seen a substantial increase over a few years, there is still no decrease in dropout rates. This study provides clear evidence that school dropout is a persisting issue in India affecting educational attainment progress. The results of our research indicate geospatial district-level variations in primary, upper primary, and secondary dropouts.

In addition to highlighting the geographical variations in dropout rates, this study examines the association between dropouts and promotion-repetition rates. In particular, it shows a strong negative relationship between promotion rates and school dropouts, and a positive relationship between the repetition rates and the dropouts. Based on the findings, the study recommends that India’s education policy must target the hotspot districts with high dropouts rate.

Extending the study to analyze and correlate all causal factors in hot spot districts will throw light on creating navigable pathways for comprehensive intervention strategies. In addition, the maps and tables provided in our study will provide a substantial base for policymakers to initiate conversations based on highlighted hotspots. This kind of GIS-based study can assist the first step in developing a strategy to enhance the nation’s educational infrastructure and, in turn, its economic situation.

Though the government has been consistently making efforts to improve the educational standards, the north-eastern and central districts in India need to be in a better position to provide standard education due to the high number of hotspots in this region. In the continuum to this conclusion, this study calls for rigorous preventive measures to reduce dropouts in achieving quality education. with the ultimate goal of achieving global education standards that will enable our future generations to strive and prosper more successfully on the planet.

## Supporting information

S1 Dataset (ZIP)

S1 Appendix (CSV)

## References

[1] Government of India M of E. UDISE+2019_20_Booklet.pdf [Internet]. p. 162. https://dashboard.udiseplus.gov.in

[2] Hanewicz C. Using geographic information systems to identify student retention patterns. 2007.

[3] Paper UW. Universalizing elementary education in India: Achievements and challenges Working Paper 2017–3 Universalizing Elementary Education in India Achievements and Challenges prepared for the UNRISD project New Directions in Social Policy: Alternatives for a. 2017.

[4] Saleh IA, Balakrishnan P. GIS Based Hotspot and Cold-spot Analysis for Primary Education in India. Indian J Sci Technol. 2019;12(45):01–33.

[5] Gubbels J, van der Put CE, Assink M. Risk Factors for School Absenteeism and Dropout: A Meta-Analytic Review. J Youth Adolesc. 2019 Sep;48(9):1637–67. doi: 10.1007/s10964-019-01072-5 31312979 PMC6732159

[6] Prakash R, Beattie T, Javalkar P, Bhattacharjee P, Ramanaik S, Thalinja R, et al. Correlates of school dropout and absenteeism among adolescent girls from marginalized community in north Karnataka, south India. J Adolesc. 2017 Dec;61:64–76. doi: 10.1016/j.adolescence.2017.09.007 28968543

[7] Majumder A, Mitra C. Dropout Behaviour of Children: The Case of West Bengal. Indian J Hum Dev. 2020;14(2):275–89.

[8] Banik A, Neogi D. Poverty and earning compulsions for the family have pushed children out of schools: A study in dropouts in basic education level in North-East India. Int J Soc Econ. 2015;42(10):946–58.

[9] Mitra S, Mishra SK, Abhay RK. Out-of-school girls in India: a study of socioeconomic-spatial disparities. GeoJournal [Internet]. 2022;7. doi: 10.1007/s10708-022-10579-7 35261431 PMC8895688

[10] Cobb CD. Geographic methods & policy: Using geographic information systems to inform education policy. Educ Res Q. 2003;27(1):28.

[11] Jogani C. Spatial Analysis of an Education Program and Literacy in India. Rev Reg Stud. 2021;41–63.

[12] Trinidad JE. Spatial analysis of high school dropout: The role of race, poverty, and outliers in New York City Spatial analysis of high school dropout: The role of race, poverty, and outliers in New York City. 2022.

[13] Schafer MJ, Hori M. the Spatial Dynamics of High School Dropout: the Case of Rural Louisiana. South Rural Sociol. 2006;21(1):55–79.

[14] Khan J, Shil A, Prakash R. Exploring the spatial heterogeneity in different doses of vaccination coverage in India. PLoS One. 2018;13(11):1–20. doi: 10.1371/journal.pone.0207209 30485291 PMC6261550

[15] Getis A, Ord JK. The analysis of spatial association by use of distance statistics. In: Perspectives on spatial data analysis. Springer; 2010. p. 127–45.

[16] Moran PAP. The interpretation of statistical maps. J R Stat Soc Ser B. 1948;10(2):243–51.

[17] Anselin L. Local indicators of spatial association—LISA. Geogr Anal. 1995;27(2):93–115.

[18] Clark PJ, Evans FC. Distance to nearest neighbor as a measure of spatial relationships in populations. Ecology. 1954;35(4):445–53.

[19] Cliff AD, Ord K. Spatial autocorrelation: a review of existing and new measures with applications. Econ Geogr. 1970;46(sup1):269–92.

[20] Bridgeland JM, Dilulio JJ Jr, Balfanz R. The high school dropout problem: Perspectives of teachers and principals. Educ Dig. 2009;75(3):20.

[21] Nayak KV, Kumar R. In Pursuit of Education: Why Some Tribal Girls Continue and Others Dropout of Schools in Rural India? J Hum VALUES. 2022 May;28(2):129–42.

[22] Singh R, Mukherjee P. ‘Whatever she may study, she can’t escape from washing dishes’: gender inequity in secondary education–evidence from a longitudinal study in India. Compare [Internet]. 2018;48(2):262–80. Available from: doi: 10.1080/03057925.2017.1306434

[23] Siddhu G. Who makes it to secondary school? Determinants of transition to secondary schools in rural India. Int J Educ Dev. 2011 May;31(4, SI):394–401.

[24] Paul R, Rashmi R, Srivastava S. Does lack of parental involvement affect school dropout among Indian adolescents? evidence from a panel study. PLoS One. 2021 May;16(5). doi: 10.1371/journal.pone.0251520 33970973 PMC8109829

[25] Peter S, Raman KJ, Ravilochanan P. School Dropouts of SC and ST Students in Chennai Corporation Schools. INDIAN J Soc Work. 2007 Apr;68(2):248–58.

[26] Marphatia AA, Reid AM, Yajnik CS. Developmental origins of secondary school dropout in rural India and its differential consequences by sex: A biosocial life-course analysis. Int J Educ Dev [Internet]. 2019;66(February):8–23. Available from: doi: 10.1016/j.ijedudev.2018.12.001

[27] Mohan A, Gutjahr G, Pillai NM, Erickson L, Menon R, Nedungadi P. Analysis of School Dropouts and Impact of Digital Literacy in Girls of the Muthuvan Tribes. In: 2017 5th IEEE International Conference on Moocs, Innovation and Technology in Education (MITE). 2017. p. 72–6.

[28] Nangia Sagarika, Jhilmil Anurag IG. A Machine Learning Approach to Identity the Students at the Risk of Dropping Out of Secondary Education in India. Adv Intell Syst Comput [Internet]. Available from: doi: 10.1007/978-981-15-2475-2_51

[29] Dobbie W, Fryer RG Jr. Are high-quality schools enough to increase achievement among the poor? Evidence from the Harlem Children’s Zone. Am Econ J Appl Econ. 2011;3(3):158–87.

[30] Freeman J, Simonsen B. Examining the impact of policy and practice interventions on high school dropout and school completion rates: A systematic review of the literature. Rev Educ Res. 2015;85(2):205–48.

